# Two-Dimensional Materials for Wide-Bandgap and Ultrawide-Bandgap Semiconductor Integration: Epitaxy, Interfaces, and Optoelectronic Devices

**DOI:** 10.3390/mi17101171

**Published:** 2026-10-08

**Authors:** Xitong Hong, Zhiqiang Xue, Mao Jia, Tong Bu, Xinpei Duan

**Affiliations:** 1 State Key Discipline Laboratory of Wide Bandgap Semiconductor Technology, School of Microelectronics, Xidian University, Xi’an 710071, China; hongxitong@hnu.edu.cn; 2 Nanjing Electronic Devices Institute, Nanjing 210016, China; tongbu@hnu.edu.cn; 3 China Electronics Technology Group Corporation 58th Research Institute, Wuxi 214072, China; dxp@hnu.edu.cn

**Keywords:** two-dimensional materials, wide-bandgap semiconductors, ultrawide-bandgap semiconductors, heterogeneous integration, van der Waals epitaxy

## Abstract

Wide-bandgap (WBG) and ultrawide-bandgap (UWBG) semiconductors are key materials for high-power electronics, radio-frequency electronics, and ultraviolet optoelectronics. However, heterogeneous integration remains challenging because conventional heteroepitaxy is constrained by lattice mismatch and differences in thermal expansion coefficients, while interface states and non-ideal metal–semiconductor contacts can degrade carrier transport and device performance. Two-dimensional (2D) materials offer an important alternative approach to heterogeneous integration. Their dangling-bond-free surfaces and van der Waals interactions relax lattice-matching requirements and facilitate membrane release, while their tunable electronic properties enable control over band alignment and interfacial carrier transport. This review examines the integration of 2D materials with WBG and UWBG semiconductors, focusing on epitaxial growth, interface physics, optoelectronic devices, and scalable integration. Representative 2D material-assisted epitaxial modes include remote, pinhole, and van der Waals epitaxy. Mixed-dimensional heterostructures are discussed in terms of their interfacial electronic properties, contact engineering, passivation strategies, and thermal transport. Photodetection represents an important optoelectronic application of these heterostructures. Taken together, these advances highlight the growing importance of 2D materials in WBG and UWBG integration and point to broader opportunities in future electronics.

## 1. Introduction

Wide bandgap (WBG) and ultrawide bandgap (UWBG) semiconductors are key materials for high-power electronics, radio frequency electronics, and ultraviolet optoelectronics [1,2,3,4,5,6]. Representative materials such as GaN, AlN, SiC, diamond, and *β*-Ga_2_O_3_ offer different combinations of wide bandgaps, high breakdown fields, high electron saturation velocities, and thermal stability [1,3,5,7]. Heterogeneous integration combines these material advantages with complementary electronic, optical, and thermal functions [8,9,10]. However, integration of dissimilar semiconductors is constrained by lattice and thermal expansion mismatch, which can generate strain, dislocations, and cracking during growth and processing [11,12,13]. Interfacial dangling bonds, intermixing, and defect states can promote carrier trapping and recombination [9,14]. Nonideal contacts and Fermi-level pinning further limit carrier injection and transport [15,16,17,18]. At larger scales, substrate availability and process compatibility impose additional constraints [8,9,10]. These interfacial and processing constraints make interface formation and interfacial transport central to WBG and UWBG heterointegration.

Two-dimensional (2D) materials provide an alternative route for integrating dissimilar materials. Their atomically thin geometry and largely dangling bond free basal planes favor van der Waals coupling across dissimilar surfaces [19,20,21]. This reduces reliance on direct covalent bonding and relaxes lattice-matching constraints. In graphene-assisted remote epitaxy, weak interfacial coupling enables membrane release after growth [21]. The tunable work functions, carrier densities, and band structures of 2D materials also allow control over band alignment, contact properties, and interfacial carrier transport [22,23]. Chemically stable 2D materials such as h-BN can further passivate exposed WBG surfaces [24]. Realizing these functions requires precise control of interface quality, 2D layer integrity, defects, and interfacial coupling.

In this review, we organize the discussion around five functional roles of 2D materials in WBG and UWBG semiconductor integration. These roles include epitaxial templating, membrane release, band and contact engineering, surface passivation, and thermal management. They connect material growth and interface formation with device operation and scalable processing. We then discuss how 2D/WBG and 2D/UWBG interfaces form and how their electronic and thermal properties govern carrier and heat transport. We compare representative integration routes and examine how interface engineering influences optoelectronic device operation. We also discuss the challenges of preserving interface quality and function during scalable processing and long-term operation, together with future directions for 2D/WBG and 2D/UWBG integration.

## 2. Functional Roles of 2D Materials in WBG and UWBG Semiconductor Integration

Integrating 2D materials, including graphene, h-BN, and transition-metal dichalcogenides (TMDs), with WBG and UWBG semiconductors introduces atomically thin interfaces with distinct structural and electronic properties [25,26]. Their dangling-bond-free surfaces and weak vdW interactions modify interfacial bonding [27,28,29,30], while their tunable electronic properties enable control over charge transfer and band alignment [31,32]. Their mechanical compliance also facilitates strain management [33,34]. These characteristics allow 2D materials to play active roles, from epitaxial synthesis to device operation [35,36,37,38].

**Figure 1:**
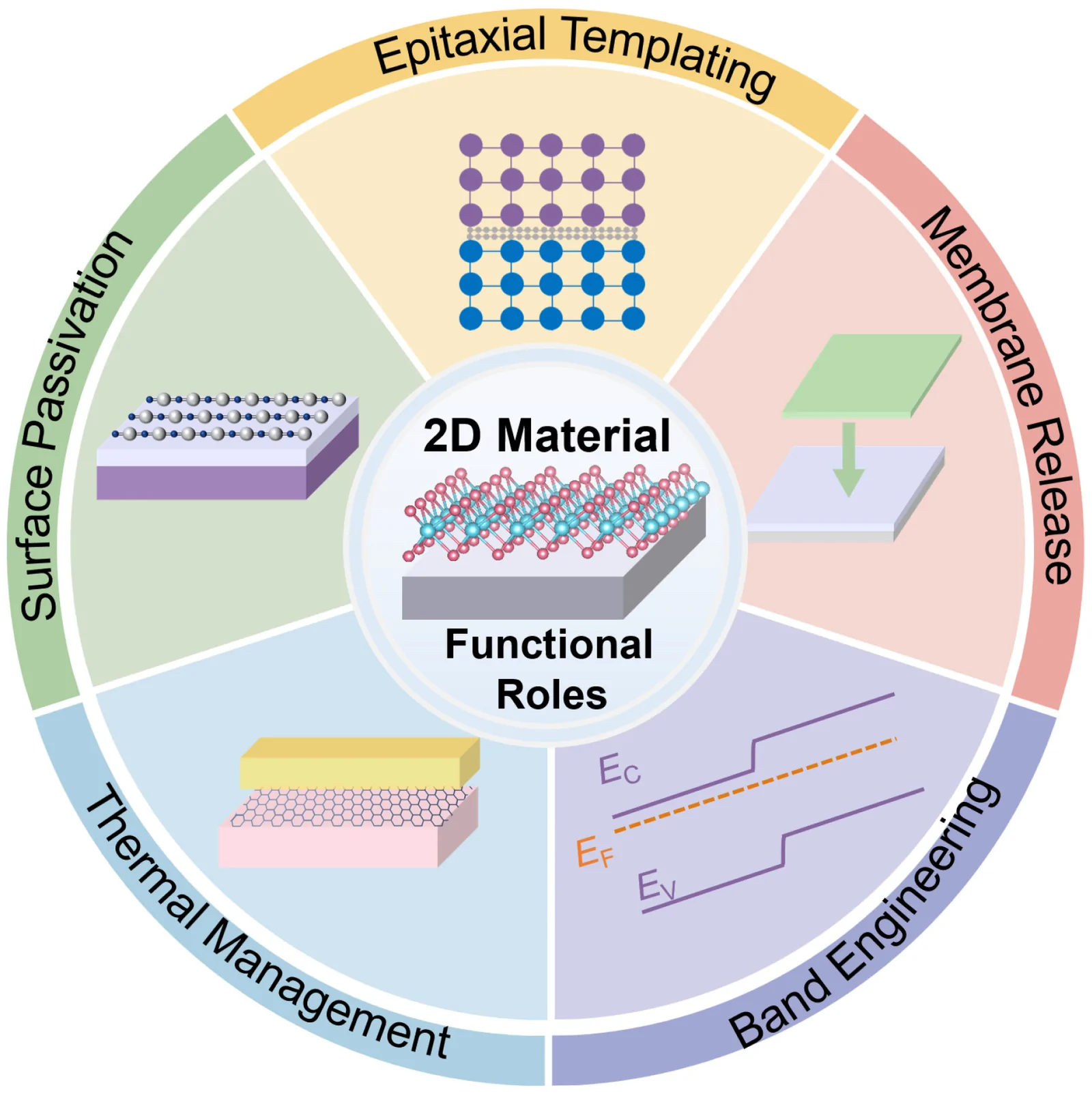
Functional roles of 2D materials in WBG and UWBG semiconductor integration.

In this review, these functions are grouped into five interconnected roles: epitaxial templating, membrane release, band and contact engineering, surface passivation, and thermal management (Figure 1). Together, they span the integration process from crystal growth and membrane handling [39,40,41,42] to interfacial electronic control and heat dissipation [43,44]. The comparison in Table 1 also highlights the main limitations associated with these functional roles. Across the five roles, 2D materials can relax lattice-matching constraints for heteroepitaxy, enable membrane release and substrate reuse, tune Schottky barriers and band offsets, reduce surface trapping, and provide lateral heat spreading. Realizing these benefits, however, depends strongly on the quality of the 2D layer and its interfaces. Low surface energy can suppress nucleation, growth-induced damage can compromise membrane release, and excessive interlayer thickness can increase tunneling resistance. Incomplete conformal coverage can undermine surface passivation, while thermal boundary resistance can limit cross-plane heat dissipation. Interface quality and process control therefore determine how effectively these functional roles translate into practical integration benefits.

In addition to these integration roles, 2D materials possess established electronic and optoelectronic functionality. Photodetectors based on h-BN, MoS_2_, WS_2_, ReS_2_, and TiS_3_ illustrate their capabilities in electronic transport and photodetection across a broad spectral range [50,51,52,53,54]. These properties provide a basis for their further use as active components in mixed-dimensional WBG and UWBG optoelectronic devices.

## 3. 2D Material-Assisted Epitaxy and Membrane Release

**Figure 2:**
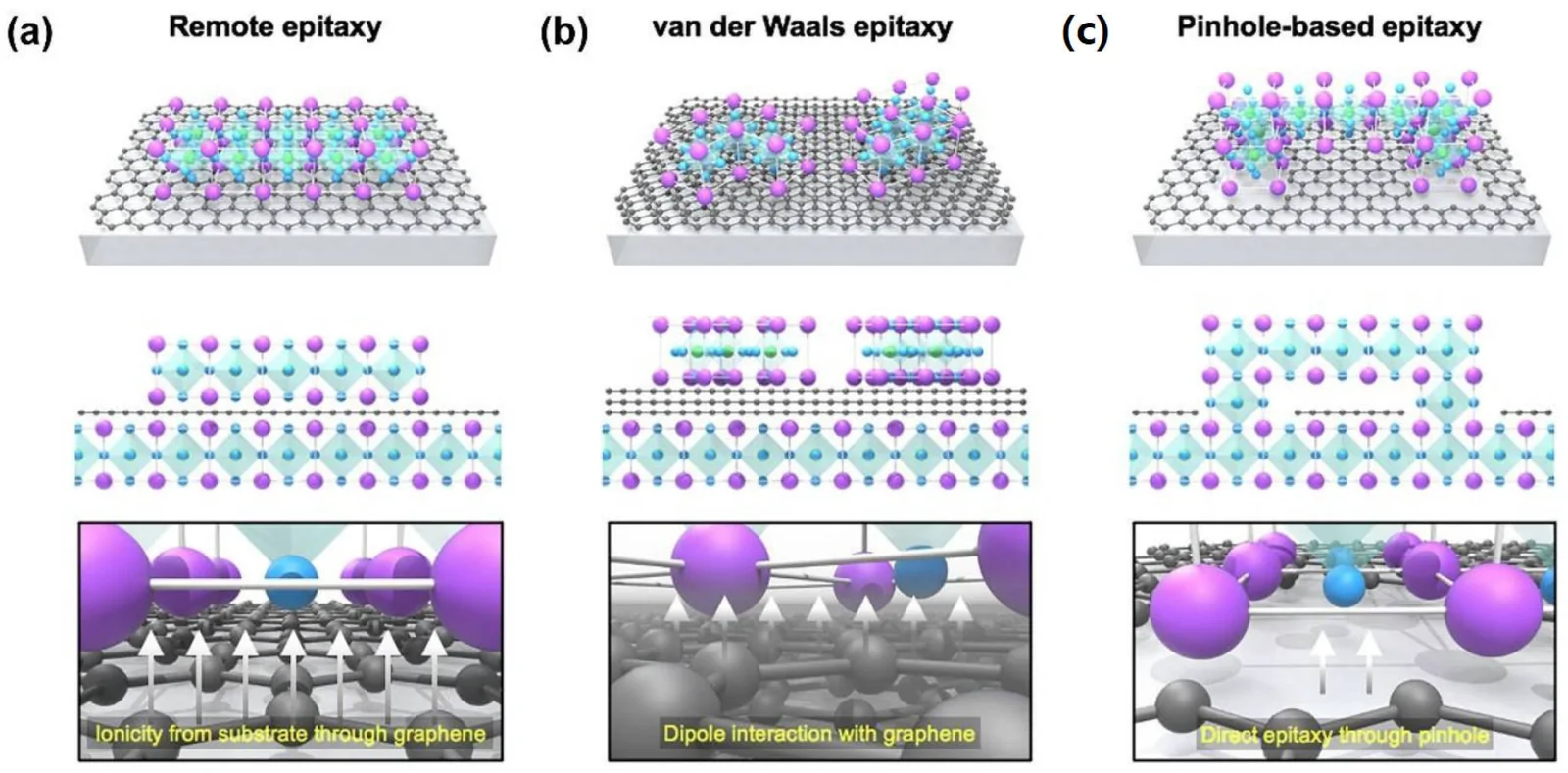
Schematic comparison of interfacial growth mechanisms: (**a**) remote epitaxy, (**b**) van der Waals epitaxy, and (**c**) pinhole epitaxy. Reprinted with permission from Ref. [30]. C.-S. Chang et al., Science Advances, https://doi.org/10.1126/sciadv.adj5379 (2023), AAAS.

The insertion of 2D layers at the growth interface alters the thermodynamics and kinetics of semiconductor epitaxy [55,56]. Weak van der Waals coupling can relax lattice-matching constraints between the growing epilayer and the substrate, whereas the low surface energy of pristine 2D surfaces can suppress nucleation [55,57,58]. These interfacial characteristics influence strain relaxation, crystal quality, and the feasibility of membrane release and substrate reuse [59,60]. In this section, 2D material-assisted epitaxy is discussed in terms of three growth modes: remote epitaxy, pinhole epitaxy, and van der Waals epitaxy [61]. Figure 2 compares their interfacial growth mechanisms.

In remote epitaxy, the partially screened electrostatic potential of the underlying substrate extends through an atomically thin 2D interlayer and guides epitaxial registry at the growth surface [29,30]. This remote interaction can orient the epilayer with respect to the underlying substrate while preserving a weakly bonded interface that facilitates subsequent exfoliation [30,62]. The strength of this interaction depends on both substrate ionicity and the screening behavior of the 2D interlayer, which varies with its thickness [29,30]. For graphene, potential fields from predominantly covalent substrates can be strongly screened by a monolayer, whereas those from more ionic substrates can extend through a few graphene layers [29]. GaN remote epitaxy and subsequent exfoliation have been demonstrated using graphene interlayers [62,63,64].

Pinhole epitaxy occurs when defects or openings in the 2D interlayer expose localized regions of the underlying substrate [65,66]. Adatoms can form localized covalent bonds at these exposed sites while retaining weaker vdW coupling over the surrounding 2D-covered regions. Such sites promote nucleation and lateral coalescence, but the resulting local bonding can hinder subsequent membrane release. Such openings may arise during processing or be introduced deliberately. Engineered graphene nanopatterns provide controlled nucleation windows that can reduce crystallographic defects in the coalesced epilayer while still enabling membrane release [67].

In van der Waals epitaxy, the 2D interlayer is thick enough to screen the substrate potential. Growth is then governed primarily by weak interactions with the 2D surface rather than by exposed-substrate nucleation sites [55,61]. Because of the weak interfacial forces and low surface energy, adatoms can favor three-dimensional island nucleation and Volmer–Weber-type growth rather than immediate continuous-film formation [55,68]. To improve nucleation and surface wetting, low-temperature seed or buffer layers can be introduced before high-temperature WBG growth. For example, ZnO-coated graphene templates have yielded transferable GaN layers [35]. In a complementary approach, increasing the growth temperature improves the crystallinity and surface morphology of GaN films grown on MoS_2_ [69]. Alternatively, GaN quantum dots can be grown directly on h-BN with high optical quality and weak substrate coupling [70].

Distinguishing remote epitaxy from pinhole-assisted growth requires more than crystallographic registry. Remote epitaxy and pinhole-assisted growth can both produce an epilayer aligned with the underlying substrate [61,65,66]. The continuity of the 2D interlayer at the growth interface should therefore be examined. Local defects or exposed substrate regions can provide direct nucleation sites and still produce substrate-related epitaxial alignment [65,66,67]. Variations in growth behavior with interlayer thickness and defect configuration can further help distinguish the dominant mechanism [29,61]. Remote, pinhole, and van der Waals epitaxy differ in both their nucleation mechanisms and the nature of the resulting interfacial bonding. Weak interfacial coupling in remote and van der Waals epitaxy facilitates mechanical exfoliation, whereas localized covalent bonding in pinhole epitaxy may hinder clean release [60,61,65,66]. Release further depends on the integrity of the 2D interlayer during WBG epitaxy [60,62,64]. For GaN growth on graphene, elevated growth temperatures or H_2_ carrier gas have been reported to damage monolayer graphene and impair exfoliation [62]. Graphene degradation near defects in the growth template can also lead to release failure [64]. With a sufficiently weak interface, mechanical exfoliation enables separation of the epitaxial membrane. GaN remote epitaxy provides a representative example [62,63]. A common release strategy uses a stressed overlayer, such as a metal film, to provide the driving force for delamination along the weak interface [71]. The applied strain energy must exceed the interfacial adhesion energy without fracturing the epilayer or substrate. This mechanical approach can avoid wet etching, sacrificial layer oxidation, or laser lift-off. After release, transfer cleanliness becomes a separate concern because polymer scaffolds can leave residues at the interface [72].

As summarized in Table 2, the three modes differ in their interfacial bonding, crystal quality, and membrane release. These differences depend on the properties and integrity of the 2D interlayer, along with the substrate/2D interaction and the growth conditions [29,30,61]. Together, the modes establish the structural basis for heterogeneous integration. The electronic properties of the resulting interfaces, in turn, govern carrier transport and device operation. The next section turns to the interface physics of 2D/WBG and 2D/UWBG heterostructures.

## 4. Interface Physics of 2D/WBG and 2D/UWBG Heterostructures

Integration of 2D materials with WBG and UWBG semiconductors forms mixed-dimensional van der Waals heterojunctions in which direct covalent bonding across the interface is not required. This relaxes the lattice-matching constraints of conventional heterostructures [73,74]. The electronic properties of these interfaces are set by the band alignment [75,76], interface dipoles [77,78], and interface trap states. These factors in turn govern charge transfer, carrier transport, and tunneling across the interface [79].

### 4.1. Band Alignment and Carrier Separation

**Figure 3:**
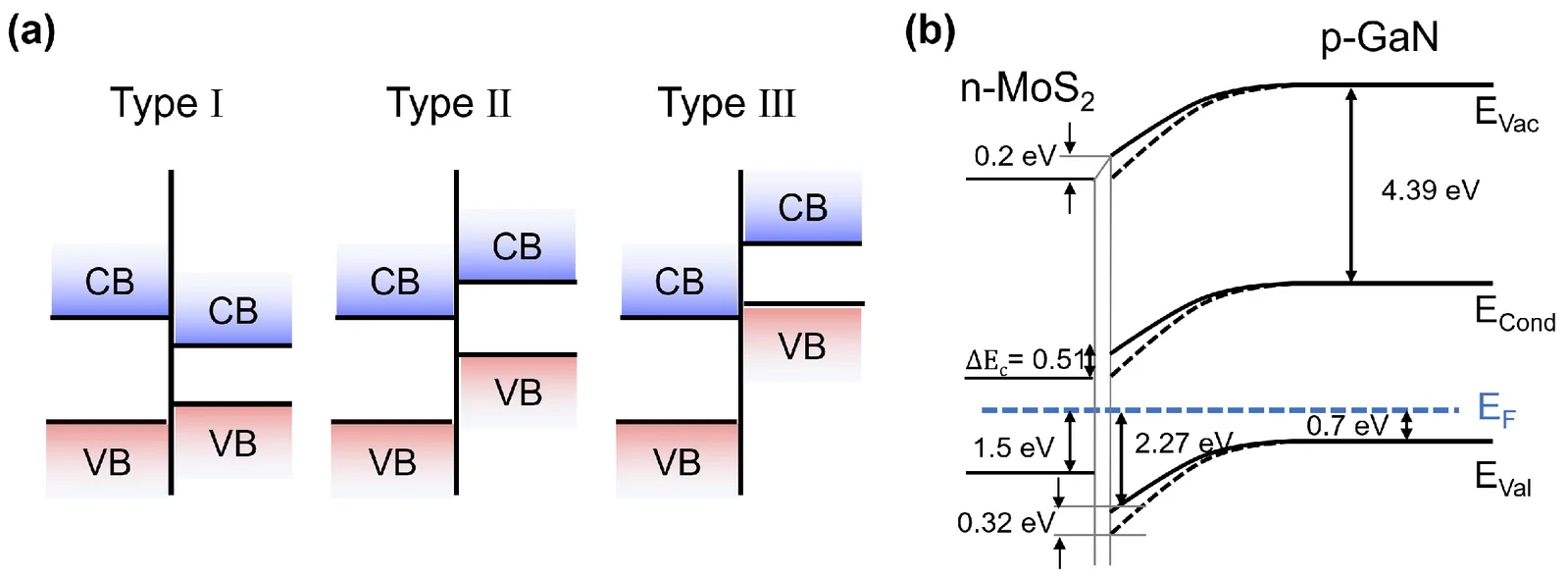
Band alignment in mixed-dimensional heterojunctions. (**a**) Idealized type-I, type-II, and type-III band configurations. (**b**) Experimentally determined interfacial band diagram of a MoS_2_/GaN(0001) heterojunction, showing the interface dipole and band bending after Fermi-level equilibration. Panel (**b**) is reprinted with permission from Ref. [80]. Copyright 2017 by the American Physical Society.

The electronic transport and optoelectronic properties of mixed-dimensional heterojunctions are strongly influenced by their interfacial band alignment [75,76]. Depending on the relative band-edge positions of the constituent materials, heterojunctions are commonly classified as straddling type-I, staggered type-II, or broken-gap type-III. Figure 3 illustrates these three idealized configurations together with the experimental interfacial band diagram of a representative MoS_2_/GaN heterojunction [80]. In ideal type-I alignment, both the conduction band minimum and valence-band maximum are located in the narrower-bandgap material, favoring carrier confinement. In ideal type-III alignment, the conduction band minimum of one material lies below the valence-band maximum of the other, allowing band-to-band tunneling. These idealized band configurations describe the equilibrium band-edge arrangement but do not fully represent carrier transport during device operation. Interfacial charge redistribution, interface states, polarization, illumination, and applied bias can modify the local potential and carrier-transfer pathways. Among these configurations, staggered type-II band alignment favors the spatial separation of photogenerated electrons and holes [81]. In a type-II heterojunction, the conduction band minimum and valence band maximum of one material are both energetically higher, or both lower, than the corresponding band edges of the adjacent material. Upon junction formation, Fermi-level equilibration induces interfacial charge redistribution and band bending [80]. Under illumination, the band offsets favor electron transfer toward the material with the lower conduction band minimum and hole transfer toward the material with the higher valence band maximum. Such spatial separation reduces the probability of electron–hole recombination and increases the number of carriers available for collection. Carrier transport and device response also depend on the magnitudes of the conduction- and valence- band offsets (*ΔE_C_* and *ΔE_V_*), carrier mobility, interface-state density, and applied bias. Large band offsets may hinder carrier extraction, while interfacial contamination and defect states introduce additional transport barriers and recombination pathways. Carrier extraction therefore depends on the specific band offsets and interface quality of each heterojunction.

Representative 2D/WBG and 2D/UWBG systems include TMD heterojunctions with *β*-Ga_2_O_3_ and GaN [78,82,83,84]. In these studies, X-ray photoelectron spectroscopy was used to determine the valence-band offsets, whereas the *ΔE_C_* were derived from the *ΔE_V_* and the constituent bandgaps. For MoS_2_/*β*-Ga_2_O_3_, Ref. [78] reported a *β*-Ga_2_O_3_ bandgap of 4.70 ± 0.10 eV and used 1.40 ± 0.10 eV for few-layer MoS_2_. The conduction-band offset was calculated as $\Delta E_{C}=E_{g}^{\beta \text{-}{\mathrm{Ga}}_{2}O_{3}}-E_{g}^{{\mathrm{MoS}}_{2}}-\Delta E_{V}$. Electron-affinity values were not used in this XPS-based determination. The measured band offsets change with interface treatment in MoS_2_/*β*-Ga_2_O_3_ [78] and with GaN crystal orientation in ReSe_2_/GaN [84]. First-principles calculations further indicate a thickness dependence of the band offsets in MoS_2_/*β*-Ga_2_O_3_ [82]. Table 3 summarizes the reported alignment types and band offsets for representative 2D/WBG and 2D/UWBG heterostructures together with the corresponding interface and structural conditions.

### 4.2. Interfacial Dipoles and Band Offsets

In real mixed-dimensional heterojunctions, band offsets can depart from the values predicted by the Anderson electron-affinity model [75,77]. This is evident in MoS_2_/*β*-Ga_2_O_3_, where N_2_ plasma treatment of the *β*-Ga_2_O_3_ surface changed *ΔE_C_* from 0.43 ± 0.10 to 0.68 ± 0.10 eV and *ΔE_V_* from 2.87 ± 0.10 to 2.62 ± 0.10 eV [78]. The authors associated this change with an interfacial dipole arising from charge transfer across the interface [78]. Band offsets also vary with the crystal orientation of the WBG semiconductor. For ReSe_2_/GaN, different *ΔE_V_* were measured for polar (0001), semipolar (11−22), and nonpolar (11−20) GaN, with corresponding changes in the derived *ΔE_C_* [84]. Despite these differences, all three interfaces retain type-II alignment [84]. The orientation dependence has been discussed in relation to the different polarization characteristics of these GaN surfaces [84].

### 4.3. Trap States and Persistent Photoconductivity Suppression

Band alignment describes the equilibrium electronic structure, whereas the temporal photoresponse is affected by carrier trapping. *β*-Ga_2_O_3_ provides a well-studied example. In a detector based on *β*-Ga_2_O_3_ grown by pulsed laser deposition (PLD), the photocurrent showed a slow decay time of 1.58 s after 250 nm illumination was switched off [85].

Defect spectroscopy has identified a broad distribution of deep levels in *β*-Ga_2_O_3_. Thermally stimulated current measurements on PLD-grown films identified trap activation energies from 0.16 to 1.03 eV, with the 1.03 eV level being the dominant trap and a possible contributor to persistent photoconductivity [85]. Optically and thermally stimulated defect spectroscopy further revealed several defect states across the *β*-Ga_2_O_3_ bandgap [86]. Self-trapped holes represent a distinct contribution to the photoresponse. Photocapacitance measurements in *β*-Ga_2_O_3_ Schottky diodes associated hole self-trapping with photoconductive gain [87]. These trapping processes have different physical origins. Deep defect levels originate from defects in the semiconductor, whereas carrier self-trapping involves carrier localization accompanied by lattice relaxation. Interface trap states arise at material interfaces and affect charge capture and interfacial transport. Interface states also affect the temporal response of WBG photodetectors. In W/graphene/*β*-Ga_2_O_3_ Schottky photodiodes, insertion of monolayer graphene reduced the reported rise and delay times from 2000 and 3000 ms to 139 and 200 ms, respectively [47]. The same study reported a lower interfacial state density after graphene insertion [47]. Beyond the heterojunction interface, 2D materials are used to modify metal contacts, passivate exposed semiconductor surfaces, and contribute to thermal management in WBG and UWBG devices.

## 5. 2D Materials for Contact Engineering, Surface Passivation, and Thermal Management

**Figure 4:**
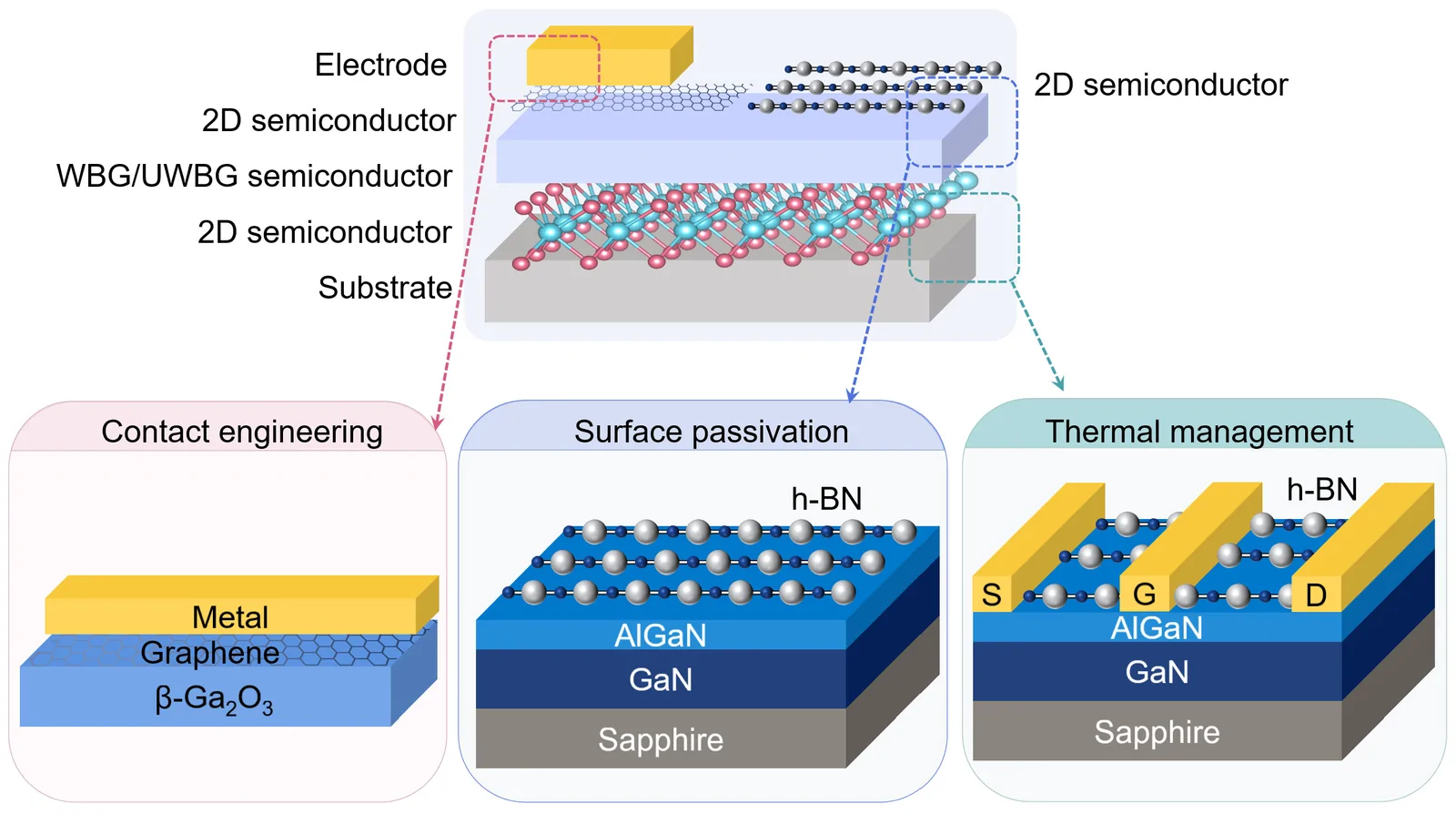
2D materials for contact engineering, surface passivation, and thermal management in WBG and UWBG electronics.

In WBG and UWBG devices, metal contacts, surface states, and self-heating impose distinct electrical and thermal limitations. 2D materials have been used at these interfaces to improve carrier injection, passivate surface traps, and facilitate heat dissipation [38,44,47]. Figure 4 highlights representative examples of contact engineering, surface passivation, and thermal management.

At metal–semiconductor contacts, Fermi-level pinning reduces the sensitivity of the Schottky barrier height to the metal work function [88,89]. Calculations for 2D metal/2D semiconductor van der Waals junctions showed that suppressing metal-induced gap states weakens Fermi-level pinning and allows more effective tuning of the Schottky barrier [90]. Experimental studies have also approached the Schottky–Mott limit [91]. In W/graphene/*β*-Ga_2_O_3_ Schottky photodiodes, insertion of monolayer graphene reduced the leakage current and ideality factor, with a reported Schottky barrier height of approximately 0.53 eV [47]. These changes were attributed to a lower interfacial state density and weaker Fermi-level pinning [47]. As a direct contact electrode, graphene showed different contact behavior on AlGaN/GaN depending on the AlGaN surface microstructure, with Schottky behavior on uniform AlGaN and Ohmic behavior on V-defect-rich AlGaN [37]. These two configurations involve different contact interfaces. In metal/graphene/semiconductor structures, graphene modifies the metal–semiconductor contact as an interfacial layer, whereas direct graphene electrodes form a graphene–semiconductor contact that governs carrier injection. Therefore, 2D materials can be introduced as interlayers at metal–semiconductor interfaces in WBG and UWBG devices, while conductive 2D materials may also act directly as contact electrodes [37,47].

At exposed semiconductor surfaces, 2D dielectrics such as h-BN have been used as passivation layers. In AlGaN/GaN high-electron-mobility transistors (HEMTs), trapping at surface states contributes to current collapse and current dispersion [92,93,94]. h-BN has a dangling-bond-free surface and can form a weakly interacting van der Waals interface with AlGaN [38]. In an h-BN/AlGaN/GaN structure grown by metal organic chemical vapor deposition (MOCVD), an approximately 2.5 nm h-BN layer formed an atomically sharp interface, while the passivated HEMTs exhibited suppressed current collapse [38]. The corresponding HEMTs reached a cutoff frequency of 28 GHz and a maximum oscillation frequency of 88 GHz [38]. Self-heating is an important thermal limitation in WBG and UWBG devices. The relatively low thermal conductivity of *β*-Ga_2_O_3_ limits heat removal [95], while localized heating in GaN transistors degrades device performance and reliability [96]. For thermal management, 2D materials such as graphene and h-BN can assist lateral heat spreading because of their high in-plane thermal conductivities [97,98]. However, efficient in-plane heat spreading does not necessarily ensure efficient cross-plane heat removal. Cross-plane transport also depends on out-of-plane thermal conduction and the thermal boundary resistance at heterogeneous interfaces. h-BN has also been proposed as a release layer for transferring GaN HEMTs to diamond [44]. In the corresponding simulation, replacing the SiO_2_/GaN/sapphire structure with an h-BN/GaN/diamond structure reduced the peak lattice temperature from 507 to 372 K and lowered the total thermal resistance by about fivefold [44]. Independent measurements on GaN-on-diamond transistors using three-dimensional Raman thermography showed a 40% lower peak channel temperature than comparable GaN-on-SiC devices [99]. The same study identified the GaN/diamond interface as a major source of thermal resistance [99]. Thermal boundary resistance at heterogeneous interfaces can limit heat removal even when the adjoining materials have high thermal conductivity [99,100].

Contact engineering, surface passivation, and thermal management address different electrical and thermal limitations in WBG and UWBG devices. In mixed-dimensional optoelectronic heterostructures, 2D materials also contribute to light absorption, carrier transport, and charge collection.

## 6. Optoelectronic Devices Based on 2D/WBG and 2D/UWBG Heterostructures

**Figure 5:**
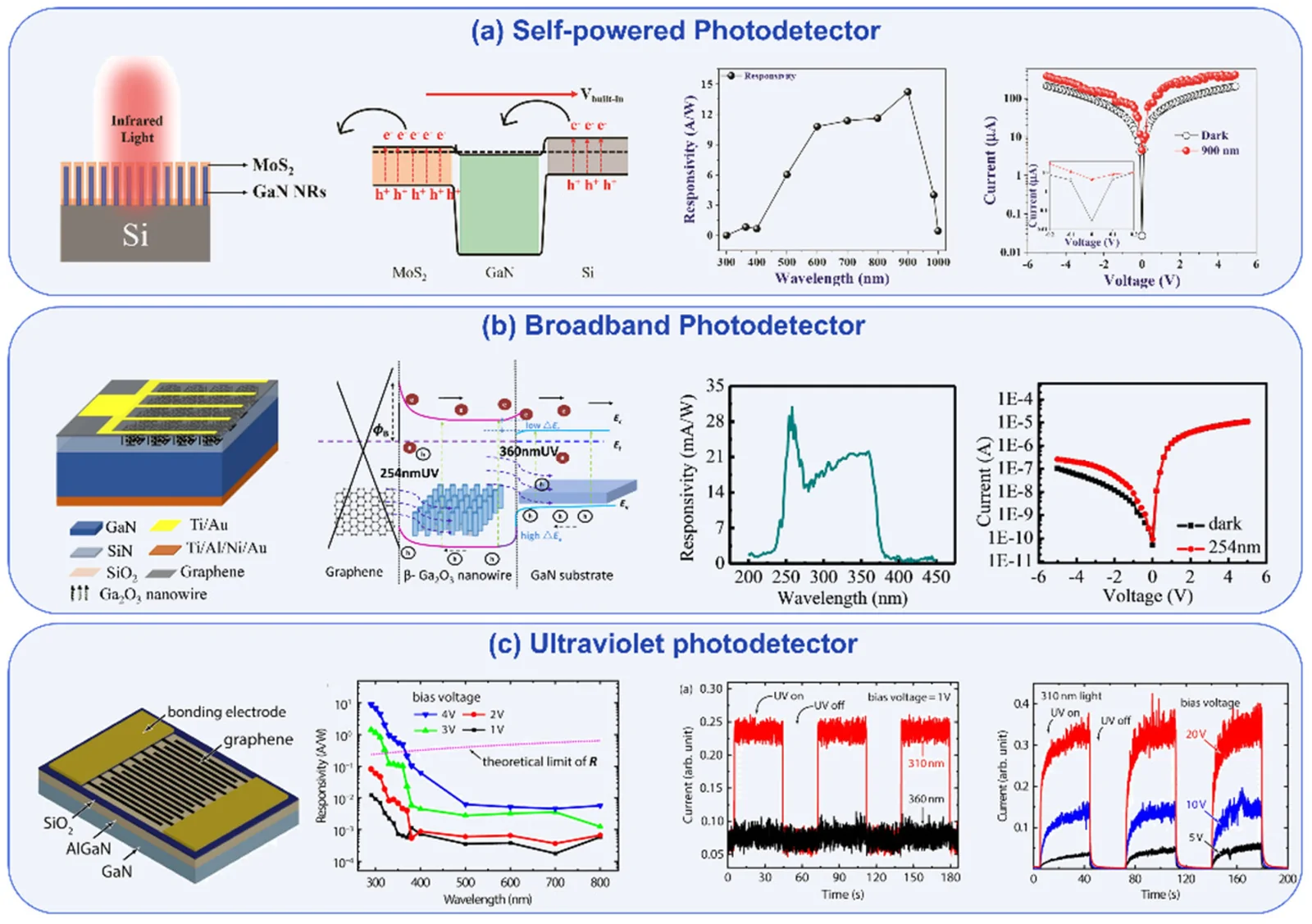
Representative photodetectors based on 2D/WBG and 2D/UWBG heterostructures. (**a**) Self-powered MoS_2_/GaN nanorod/Si photodetector showing the device structure, band alignment, spectral responsivity, and zero-bias photovoltaic response. Adapted from Ref. [102], licensed under CC BY 3.0. (**b**) Broadband ultraviolet graphene/*β*-Ga_2_O_3_ nanowires/GaN photodetector showing the device structure, band alignment, spectral responsivity, and current–voltage characteristics. Adapted from Ref. [103], licensed under CC BY 4.0. (**c**) Graphene/AlGaN/GaN ultraviolet photodetector showing the device configuration, bias-dependent spectral responsivity, and transient photoresponse. Adapted from Ref. [104], licensed under CC BY 4.0.

WBG and UWBG semiconductors are established materials for solar-blind and DUV photodetection, but several device-level limitations remain. Photoconductive operation often requires an external bias, and the large bandgaps of these absorbers limit sensitivity at longer wavelengths. Metal electrodes can reduce optical transmission, while polarization-resolved detection commonly requires external polarization optics. The integration of 2D materials introduces additional optical and electrical functions in these devices [101,102,103,104,105]. The device response depends on interfacial carrier transport and on the roles of the constituent materials. Depending on the device architecture, the 2D component can act as a transparent electrode, optical absorber, carrier-transport layer, or band modulator [50,51,52,53,54]. Figure 5 summarizes representative self-powered, broadband, and ultraviolet (UV) photodetectors.

Self-powered photodetection relies on the built-in field of a heterojunction to separate and collect photogenerated carriers without external bias. In the MoS_2_/GaN nanorod/Si device shown in Figure 5a, MoS_2_ contributes to optical absorption, while the vertically aligned GaN nanorods enhance light harvesting through their geometry [102]. The Si substrate further contributes to optical absorption and carrier collection. At zero bias, carrier separation is driven by the built-in potential across the heterojunction. Under this condition, the device exhibits a spectral response from 300 to 1000 nm and reaches a maximum responsivity of approximately 14.2 A/W at 900 nm.

Broadband UV response in 2D/WBG and 2D/UWBG photodetectors can arise from complementary absorption in semiconductors with different bandgaps. In the graphene/*β*-Ga_2_O_3_ nanowires/GaN photodetector shown in Figure 5b, graphene serves as a transparent electrode, whereas the *β*-Ga_2_O_3_ nanowires and GaN substrate provide complementary UV absorption [103]. *β*-Ga_2_O_3_ dominates the response near 258 nm, with GaN extending the detection range toward 360 nm. At the *β*-Ga_2_O_3_/GaN interface, the small conduction-band offset facilitates electron transport, whereas the larger valence-band offset restricts hole transport. The device responds over 248–368 nm and reaches a peak responsivity of approximately 30.8 mA/W at 258 nm under a bias of −5 V.

In nitride UV photodetectors, graphene may serve as a transparent electrode while also influencing bias-dependent carrier transport at the semiconductor interface. In the graphene/AlGaN/GaN device shown in Figure 5c, graphene forms interdigitated electrodes on the AlGaN/GaN heterostructure. The graphene/AlGaN contact shows Schottky-like behavior, while the underlying heterostructure supports a 2D electron gas [104]. At low bias, the device operates mainly in the photovoltaic regime and shows a fast temporal response. Above the threshold voltage, the graphene/AlGaN barrier is overcome, establishing a current path through the two-dimensional electron gas and giving rise to photoconductive gain. The responsivity reaches 10.9 A/W at 5 V under 310 nm illumination. The higher responsivity in this regime is accompanied by a slower transient response and increased dark current.

The examples in Figure 5 show that the role of the 2D component varies with device architecture in WBG and UWBG photodetectors. Table 4 extends this comparison to representative 2D/WBG and 2D/UWBG photodetectors and summarizes their spectral ranges and key performance metrics.

Across the device architectures summarized in Figure 5 and Table 4, the optoelectronic response depends strongly on how the 2D material is incorporated into the heterostructure. Device performance is therefore governed by the functional role of each component, the optical response of the constituent semiconductors, and interfacial carrier transport. Direct comparison with otherwise similar devices lacking the 2D component remains limited, making it difficult to distinguish the contribution of the 2D material from those of the other device components and interfaces. Extending these functions beyond individual devices requires reproducible integration, scalable processing, and stable operation.

## 7. Scalable Integration and Reliability

Extending the device architectures discussed above to larger areas requires the scalable integration of 2D/WBG and 2D/UWBG heterostructures [114]. Two broad routes are transfer assembly and direct growth. For transfer assembly, 2D materials grown by chemical vapor deposition (CVD) are transferred onto WBG or UWBG substrates [115,116]. PMMA is widely used as a support layer for wet transfer of large 2D films [117,118,119]. Electrochemical delamination provides an alternative that reduces catalyst consumption and enables reuse of the growth substrate [120]. Direct growth has been demonstrated in both growth sequences. 2D materials have been grown directly on WBG substrates, as demonstrated for MoS_2_/SiC [111]. WBG semiconductors have also been epitaxially grown on 2D interlayers [21,29,62,69]. When weak interfacial coupling is preserved, the 2D interlayer also facilitates membrane release and substrate reuse [21,29]. These integration routes impose different requirements on interface quality and process compatibility as the integration area increases.

Interface quality becomes more difficult to preserve as the integration area increases. In transfer assembly, polymer residues, trapped interfacial water, wrinkles, cracks, and transfer induced strain can disrupt carrier transport, leading to nonuniform interfacial barriers [72,115,118]. Hydrophobicity-mediated electrostatic adhesion enables polymer free wafer scale graphene transfer with low defect density [121]. Flat-to-flat stacking uses Spin-assisted dehydration and proton-assisted interface cleaning for wafer scale assembly of graphene based van der Waals structures [122]. Direct growth avoids transfer-related contamination but introduces its own thermal and chemical constraints. During GaN growth on graphene, lower growth temperatures and N_2_ carrier gas help preserve the graphene interlayer and support subsequent membrane release, whereas higher temperatures or H_2_ exposure damage graphene [62]. Low-temperature seed or buffer layers also improve nucleation on 2D surfaces, as shown by transferable GaN grown on ZnO-coated graphene [35]. For both routes, large area integration requires control of interface cleanliness, 2D layer integrity, film uniformity, and the process thermal budget. Table 5 compares representative integration strategies, their main advantages, and remaining limitations.

Scalable integration must be accompanied by stable device operation. In WBG devices, prolonged electrical stress has been reported to shift Schottky barrier height and threshold voltage through charge trapping and defect evolution [123]. Long-term stability in 2D devices can also be affected by charge trapping, defects, and environmental exposure [124]. These effects become particularly important in mixed dimensional heterostructures, where carrier transport is strongly influenced by the interface. Interface stability therefore remains an important reliability consideration for 2D/WBG and 2D/UWBG heterostructures.

Reliability assessment needs to reflect the operating conditions of the intended application. Bias stress, thermal cycling, and environmental aging are used to evaluate long-term device stability [123]. Radiation testing is additionally relevant for space and other radiation-exposed environments [125]. These assessments provide a basis for determining whether 2D/WBG and 2D/UWBG heterostructures retain stable performance during prolonged operation.

## 8. Conclusions and Outlook

The integration of 2D materials with WBG and UWBG semiconductors provides additional routes for heterogeneous integration and interface control. Weak van der Waals coupling relaxes lattice-matching constraints and facilitates membrane release, while the electronic properties of 2D materials enable modification of band alignment, contacts, and interfacial carrier transport. Their roles in surface passivation and thermal management further extend the functions of the interface beyond structural integration. Through these functions, 2D materials act as interfacial components that link structural integration with electronic and thermal control in WBG and UWBG heterostructures.

Despite these established functions, several unresolved issues still limit further development of 2D/WBG and 2D/UWBG integration. The mechanisms of 2D-assisted epitaxy require clearer distinction, particularly between remote epitaxy and growth mediated by defects or exposed substrate regions. Interfacial electronic properties also vary with defects, strain, polarization, illumination, temperature, and applied bias. Equilibrium band diagrams therefore provide only a partial description of carrier transport in operating devices. Interface engineering also involves performance tradeoffs. Interface states may enhance photogain but prolong photocurrent decay. Interlayers may reduce Fermi-level pinning but increase tunneling resistance, while thermal boundary resistance limits heat removal across heterogeneous interfaces. At larger scales, interface cleanliness, film uniformity, process compatibility, and long-term stability continue to limit reproducible integration. Continued progress in scalable processing and device reliability can broaden the use of 2D materials in WBG and UWBG semiconductor systems, supporting more diverse optoelectronic functions.

## Figures and Tables

**Table 1:** Functional roles of 2D materials in WBG and UWBG integration: mechanisms, benefits, and limitations.

Function Role	Representative 2D Materials	Physical Mechanism	Integration Benefit	Main Limitation or Trade-Off
Epitaxial templating	Graphene, h-BN, MoS_2_	Low-surface-energy vdW template [27,28]	Relaxed lattice-matching requirements for heteroepitaxy [35,36,41,42]	Low nucleation density on pristine surfaces
Membrane release	Graphene, h-BN	Weak interfacial binding [29,30]	Mechanical exfoliation and substrate reuse [39,40,45,46]	Damage or pinholes can prevent clean release
Band and contact engineering	Graphene, TMDs	Work-function tuning and dipole formation [31,32,47,48,49]	Schottky barrier and band-offset control [37,47,48,49]	Tunneling resistance rises with thickness
Surface passivation	h-BN	Dangling-bond-free crystalline surface coverage [24,38,49]	Suppressed gate leakage and surface trapping [24,38,49]	Wafer-scale transfer, conformality, and interfacial cleanliness remain challenging
Thermal management	Graphene, h-BN	High in-plane thermal conductivity and lateral heat spreading [43,44]	Reduced hot-spot temperature and improved thermal reliability [43,44]	High cross-plane thermal boundary resistance

**Table 2:** Comparison of growth mechanisms, epitaxial registry, and membrane release in remote, pinhole, and van der Waals epitaxy.

Growth Mode	Interface Bonding	Substrate-Potential Penetration	Lattice-Matching Requirement	Crystal Quality	Membrane Release and Substrate Reuse
Remote epitaxy	Predominantly vdW, guided by the substrate potential [29,30]	Strong (the Coulombic field permeates 1–3 monolayers)	Strict registry with the substrate [29,30]	High (strain transfer reduced) [21,55]	Exfoliation and reuse demonstrated [39,62,63,64]
Pinhole epitaxy	Mixed vdW coupling and localized covalent bonds at defects [65,66]	None	Partially relaxed; local registry at bonding sites [65,66]	Moderate (set by lateral coalescence) [65,66]	Exfoliation possible; reuse compromised by local bonding [65,66,67]
Van der Waals epitaxy	Predominantly vdW [55]	None (fully shielded by a thick 2D layer)	Substantially relaxed [19,55]	Moderate to high (Volmer–Weber Island coalescence) [55,68]	Readily exfoliated; reuse possible [55]

**Table 3:** Reported band alignments and band offsets in representative mixed-dimensional 2D/WBG and 2D/UWBG heterostructures.

Heterostructure System	Energy-Band Alignment	Conduction-Band Offset (*ΔE_C_*)	Valence-Band Offset (*ΔE_V_*)	Interface Treatment or Structural Note
MoS_2_/*β*-Ga_2_O_3_	type-II (staggered)	0.43 ± 0.10 eV	2.87 ± 0.10 eV	Pristine, untreated interface [78]
MoS_2_/*β*-Ga_2_O_3_	type-II (staggered)	0.68 ± 0.10 eV	2.62 ± 0.10 eV	N_2_-plasma-nitrided interface [78]
Trilayer MoS_2_/*β*-Ga_2_O_3_	type-I (straddling)	0.73 eV	2.28 eV	Thickness-dependent band evolution [82]
GaN/WSe_2_	type-II (staggered)	0.8 ± 0.15 eV	2.25 ± 0.15 eV	GaN epitaxially grown on single-layer WSe_2_ [83]
ReSe_2_/(0001) GaN	type-II (staggered)	1.51 ± 0.15 eV	3.08 ± 0.15 eV	Polar GaN (0001) [84]
ReSe_2_/(11−22) GaN	type-II (staggered)	1.05 ± 0.15 eV	2.62 ± 0.15 eV	Semipolar GaN (11−22) [84]
ReSe_2_/(11−20) GaN	type-II (staggered)	0.67 ± 0.15 eV	2.24 ± 0.15 eV	Nonpolar GaN (11−20) [84]

**Table 4:** Quantitative comparison of representative 2D/WBG and 2D/UWBG photodetectors.

Heterostructure	Role of the 2D Material	Spectral Range	Key Performance Metrics
MoS_2_/GaN nanorod/Si [102]	Optical absorption and heterojunction partner	300–1000 nm	*R* = 14.22 A/W at 900 nm; *τ*_r_/*τ*_d_ = 38.8/8.2 μs (10 kHz); 0 V
Graphene/β-Ga_2_O_3_ nanowires/GaN [103]	Transparent electrode	248–368 nm	*R* = 30.82 mA/W at 258 nm; *EQE* = 14.79%; D^*^ = 2.11 × 10^9^ Jones; *τ*_r_/*τ*_d_ = 0.38/0.31 s; −5 V
Graphene/AlGaN/GaN [104]	Transparent electrode	310/360 nm	*R* = 10.9 A/W at 310 nm (5 V); D^*^ = 6.19 × 10^11^ Jones (4 V); *τ*_r_/*τ*_d_ < 0.1 s (1 V)
MoS_2_/a-GaN [105]	Heterojunction partner	UV	*R* = 15 mA/W; D^*^ = 4.7 × 10^13^ Jones; *τ*_r_/*τ*_d_ = 4/8 ms; 0 V
Epitaxial graphene/4H-SiC [106]	Transparent conductive electrode	254–365 nm	*R* ≈ 4.2 mA/W at 311 nm; *R* ≈ 2.0 mA/W at 365 nm (5 V)
Epitaxial graphene/SiC [107]	Transparent junction layer	UV	*EQE* > 80% at 4 eV; dark current < 1 nA/cm^2^
Epitaxial graphene/n-SiC [108]	Transparent Schottky electrode	325 nm	*R* = 2.18 A/W at 325 nm; *EQE* > 800%; response speed tunable from tens of ns to ms; 0 to −5 V
MoSe_2_/WSe_2_/4H-SiC [109]	2D p–n heterojunction	532 nm	*R* = 7.17 A/W; *EQE* = 1.67 × 10^3^%; D^*^ = 5.51 × 10^11^ Jones; 10 V
Graphene/nanoporous GaN [110]	UV absorber and high-mobility transport layer	UV	D^*^ = 1.0 × 10^17^ Jones; UV–visible rejection 4.8 × 10^7^; −1.5 V
MoS_2_/4H-SiC [111]	Optical absorber and photoconductive layer	UV–visible	*R* = 5.7 A/W at 20 V; D^*^ = 3.07 × 10^10^ Jones at 20 V; *τ*_r_/*τ*_d_ = 0.58/0.33 s at 10 V
MoS_2_/GaN [112]	Optical absorber and heterojunction partner	532 nm and UV	*R* = 328 A/W and response time = 400 ms at 532 nm; *R* = 27.1 A/W and response time = 300 ms in UV
MoS_2_/GaN [113]	Visible-light absorber and heterojunction partner	280–850 nm	*R* on the order of 10^4^ A/W; *EQE* 500% higher than bare GaN

**Table 5:** Representative strategies for scalable integration of 2D/WBG and 2D/UWBG heterostructures.

Fabrication Strategy	Process Approach	Key Advantages	Remaining Challenges
Polymer-assisted wet transfer	PMMA-supported transfer of CVD-grown 2D films [117,118]	Mature process; large area compatibility	Polymer residues; wrinkles; cracks; transfer strain
Electrochemical delamination	Electrochemical release of CVD grown 2D films from metal catalysts [120]	Reduced catalyst use; substrate reuse	Mechanical damage; handling contamination
Hydrophobicity-mediated electrostatic transfer	Polymer free graphene transfer using hydrophobicity control and electrostatic adhesion [121]	Low defect density; wafer scale transfer	Limited validation beyond graphene and selected substrates
Flat-to-flat stacking	Spin-assisted dehydration and proton-assisted interface cleaning [122]	Clean interfaces; controlled stacking; wafer scale assembly	Limited compatibility with different substrates and device topographies
Direct growth of 2D materials	Direct CVD growth on WBG substrates, including MoS_2_ on SiC [111]	Avoids transfer and related contamination	Nucleation control; film uniformity; substrate compatibility
Epitaxy on 2D interlayers	WBG epitaxy on 2D interlayers by remote or van der Waals epitaxy [21,29,35,62]	Membrane release; substrate reuse	Thermal and chemical stability of the 2D interlayer

## Data Availability

No new data were created or analyzed in this study. Data sharing is not applicable to this article.
